# Etiologic and clinical characterization of community acquired gastroenteritis in adult patients in a Chilean emergency room by the FilmArray GI panel

**DOI:** 10.1371/journal.pone.0207850

**Published:** 2018-11-26

**Authors:** Carlos Valenzuela, Paulette Legarraga, Arturo Peña, Alex Arenas, Loni Berkowitz, Gigliola Ramírez, Aniela Wozniak, Patricia García, Manuel Álvarez-Lobos

**Affiliations:** 1 Department of Gastroenterology, Escuela de Medicina, Pontificia Universidad Católica de Chile, Santiago, Chile; 2 Facultad de Medicina, Universidad Católica de la Santísima Concepción, Concepción, Chile; 3 Department of Clinical Laboratories, Escuela de Medicina, Pontificia Universidad Católica de Chile, Santiago, Chile; 4 Emergency Room, Escuela de Medicina, Pontificia Universidad Católica de Chile, Santiago, Chile; Universita degli Studi di Parma, ITALY

## Abstract

Infectious diarrhea can be caused by a large number of microorganisms including bacteria virus and parasites. The clinical syndromic approach has been traditionally used to guide therapy. The aim of this study was to characterize the etiology of acute diarrhea by the FilmArray GI panel and to correlate it with its clinical presentation in an adult population presenting to the emergency room in a developing country. Material and Methods: Adult patients attending the ER due to acute diarrhea were selected. All patients included had a FilmArray GI panel performed and the clinical characteristics were recorded. Results: One hundred and ninety-nine patients were included. One hundred and eighteen (59.3%) were females. The mean age was 43 years old. Thirty three percent of the patients presented dysentery, 36.7% fever, 54.8% referred nauseas and 35.7% vomiting. Sixty three percent of the patients presented some degree of dehydration. In total, 221 microorganisms were detected of which 71.5% corresponded to bacteria (158/221), 19.9% to virus (44/221) and 8.6% to parasites (19/221). In 133 (67.0%) of 199 patients at least one microorganism was identified. Infections with more than one microorganism were detected in 27.1% of the patients. Polimicrobial infections were associated with a higher frequency of nausea (50.0% vs 32.0%, p 0.046), abdominal pain (87.0% vs 44.0%, p<0.0001) and travel history (20.0% vs 5.0%, p 0.0102). Bacterial infections occurred without a seasonal distribution with the exception of *Salmonella* sp whereas viral infections predominated during the autumn–winter months. Diarreicogenic *E*. *coli* were present in the context of a co-infection in more than 80.0% of the cases. Discussion: The use of multiplex panels has given us invaluable information regarding the epidemiology of acute diarrhea in adult. It highlighted the importance of polimicrobial infections and the frequency of diarreicogenic *E*. *coli* infections. Nevertheless, the lack of severity compared to monomicrobial infections and the usual association with other microorganisms in the latter make their clinical importance debatable.

## Introduction

Infectious diarrheal disease affects developing countries, causing serious morbidity and mortality [1–2]. The etiologic agents associated include viruses, bacteria and parasites [3]. Conventional methods are able to detect the etiologic agent in only 20.0% to 50.0% of the cases [4–9]. Recent studies have shown the added value of molecular multiplex detection of intestinal pathogens compared to conventional methods [10–14]. Application of these molecular tests has helped to recognize, for example, the relative high percentage of cases of diarrhea associated with more than one pathogen (16.0 to 32.0%) and the high frequency of viruses. The later usually is not taken in account in the medical evaluation of patients with acute diarrhea who attend to an emergency unit [10–14]. Although common opinion suggests that clinical presentation does not predict a specific etiologic agent [15,16], a usual clinical approach in developing nations is the syndromic diagnosis, consisting of distinguishing different types of stools (acute watery, persistent, and bloody diarrhea) in order to guide management [17]. With the aim to characterize the etiology of acute diarrhea and to correlate it with its clinical presentation a study was carried out in an adult population attending an emergency department by the BioFire FilmArray Gastrointestinal(GI) Panel (BioMérieux, Marcy- L´Étoile, France).

## Materials and methods

### Patients

A prospective study was carried out from January 2015 until March 2016 in the Clinical Hospital of the Pontificia Universidad Católica de Chile which is a private teaching hospital of 400 beds, located in a central area of the capital. It provides care for patients benefitting from the public and private health system. Attendance to the emergency department (ER) does not need the referral from an external center or general practitioner. Patients attending the ER or hospitalized due to acute diarrhea defined as passage of ≥ 3 unformed stools in 24 h associated to enteric symptoms, with a duration of symptoms of less than14 days, were eligible [17,18]. Inclusion criteria were: age ≥18 years, duration of the episode: ≥ 24 hours and less than 14 days and indication of a microbiological assessment based on the IDSA and/or ACG Guidelines [17, 18]. At the moment of enrollment in the ER the attendant physician filled a questionnaire with the relevant information requested: age, sex of the patient, use of antibiotics or other drugs, comorbidities, history of travel, days of symptoms, presence of nausea and vomiting, frequency of stool passages, presence of blood and if signs of dehydration were found at the physical exam. Any missing information was then completed through medical chart review or directly with the patient either by phone call or direct interview if possible. All the patients participating in the study had a stool sample processed by the FilmArray GI panel.

### FilmArray GI Panel

The stool samples were maintained at 4° to 8°C in a liquid Cary-Blair media (Remel) until processing (≤ 48 hours after sample retrieval) at the microbiology laboratory. The samples were analyzed using the FilmArray GI Panel following the manufacturer´s instructions. The FilmArray panel is an automated real time PCR platform capable of detecting 22 target pathogens in a single reaction including bacteria, virus and parasites. The pathogens included are: Enterotoxigenic *E*. *coli* (ETEC), Enteropathogenic *E*. *coli* (EPEC), Enteroaggregative *E*. *coli* (EAEC) and Shiga-like toxin producing *E*. *coli* (STEC) with specific identification of *E*. *coli* O157, Enteroinvasive *E*.*coli*/*Shigella* (EIEC), *Campylobacter* (*jejuni/coli/upsaliensis*), *Vibrio* (*parahaemolyticus/ vulnificus/ cholerae*) with special identification of *V*.*cholerae*, *Yersinia enterocolitica*, *Plesiomonas shigelloides*, *Clostridium difficile* (Toxin A/B), *Salmonella*, *Cryptosporidium*, *Cyclospora cayetanensis*, *Entamoeba histolytica*, *Giardia lamblia*, Adenovirus F40/41, Astrovirus, Norovirus GI/GII, Rotavirus A and Sapovirus (genogroups I,II,IV and V). In brief, 200 μL of the stool sample are added into the pouch along with hydrating solution. The pouch is then introduced into the FilmArray instrument. All steps (extraction, amplification and detection) are performed in the pouch without the need of further manipulation. Results are obtained after approximately one hour.

A description of the pathogens observed and their frequency was made as well as a statistical analysis between the clinical characteristics observed considering the results: positive vs negative, mono vs polimicrobial infection, the etiology of the episode (bacterial vs viral or parasitic) and immune status of the patient. Finally an analysis was performed regarding the clinical findings with specific microorganisms. For the clinical-etiological correlation, patients presenting results positive only for bacteria, virus or parasite were considered whereas mono and polimicrobial infections were considered for the other aspects analyzed. For the analysis we defined immunosuppression as the presence of HIV infection, hematological malignancy, solid organ transplant, immunosuppressive therapy and/or chronic renal failure.

### Statistical analysis

GraphPad Prism software (6.0) was used for the statistical analysis. Continual variables were analyzed using the Student´s T test, the comparison of 2 or more variables were analyzed by ANOVA whereas chi-square test or Fisher's exact test were used in the case of categorical variables.

### Ethics committee approval

This study was approved by the ethics committee of the medical school of the Pontificia Universidad Católica de Chile, number 14–418 and all patients included gave their written, informed consent.

## Results

Patient’s characteristics: A total of 207 patients were enrolled, of which 199 were included for analysis. Eight patients were excluded due to the presence of chronic diarrhea. One hundred and eighteen (59.3%) were females and 81 (40.7%) corresponded to male patients. The mean age was 43 years old with a median of 40 years (18–100) and 116 patients (58.3%) presented at least one comorbidity (hypertension 21.0%, diabetes 7.5% and hypothyroidism 6.5%). The average bowel movements frequency was 8.2 episodes per day, with 5.8 days of symptoms before seeking medical attention. Sixty-six patients (33.1%) presented dysentery, 73 (36.7%) fever, 109 (54.8%) referred nauseas and 71 (35.7%) vomiting. Eighty-four patients (42.2%) presented signs of mild, 39 (19.6%) moderate and 4 (2.0%) severe dehydration and 92 patients (46.2%) required hospitalization.

### Etiological agents detected by the FilmArray GI panel

**Fig 1:**
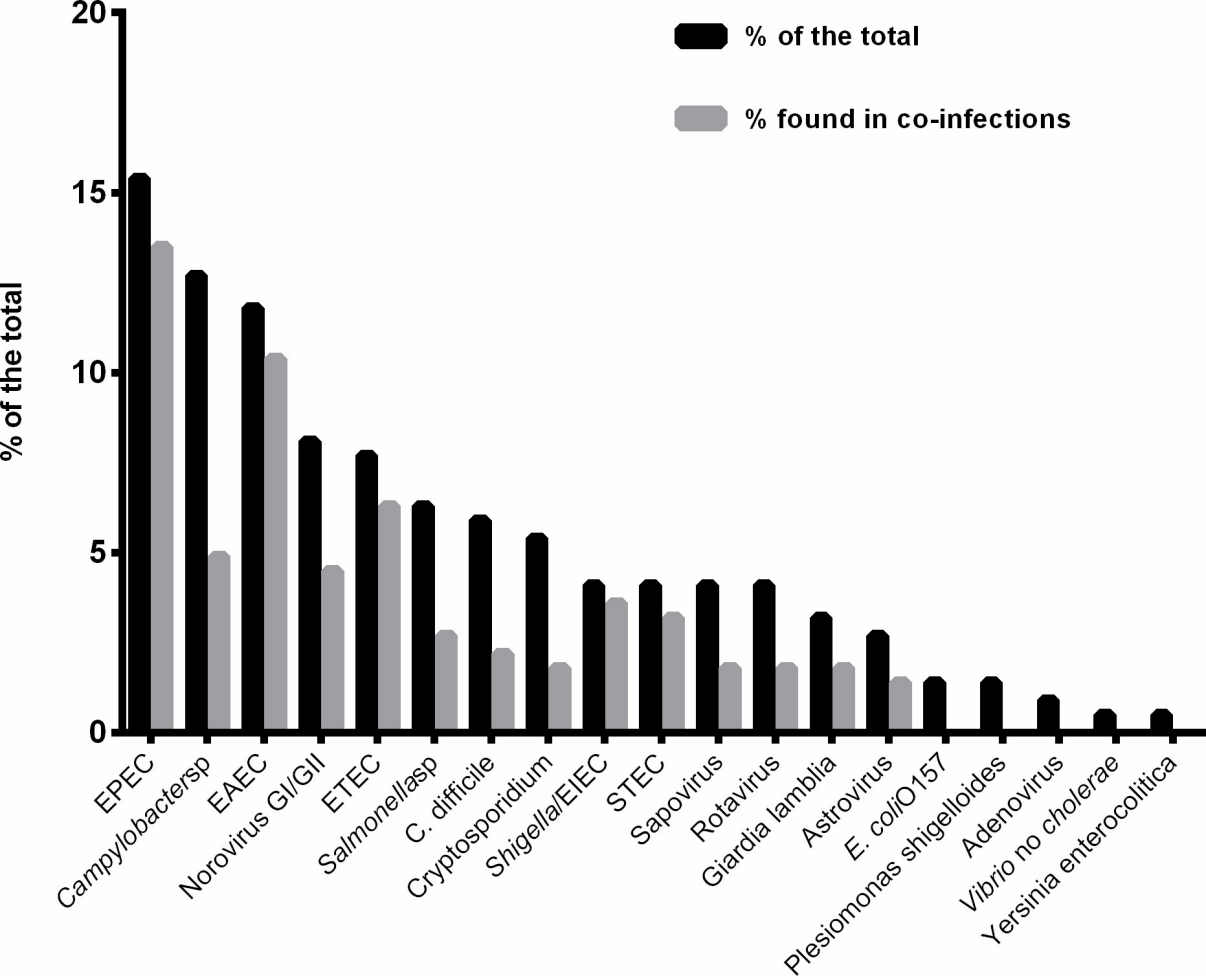
Distribution of microorganisms in order of frequency and percentage found in the context of a co-infection. EPEC: enteropathogenic *E*. *coli*, EAEC: enteroaggregative *E*. *coli*: ETEC: enterotoxigenic *E*. *coli* EIEC: enteroinvasive *E*. *coli*, STEC: shiga-toxin *E*. *coli*.

Of the 199 samples analyzed corresponding to 199 patients, at least one microorganism was identified in 133 (67.0%) patients. One microorganism was detected in 39.7% of the samples (79/199), two microorganisms in 16.6% (33/199), 3 microorganisms in 7.0% (14/199) with 4 or more microorganisms detected in 3.5% (7/199) of the samples. In total, 221 microorganisms were detected of which 71.5% corresponded to bacteria (158/221), 19.9% to virus (44/221) and 8.6% to parasites (19/221). The pathogen´s distribution in order of frequency was: EPEC 34 (15.4%), *Campylobacter* spp. 28 (12.7%), EAEC 26 (11.8%), Norovirus GI/GII 18 (8.1%), ETEC 17 (7.7%), *Salmonella* spp. 14 (6.3%), *Clostridium difficile* toxin A/B 13 (5.9%), *Cryptosporidium* 12 (5.4%), *Shigella*/EIEC 9 (4.0%), STEC 9(4.0%), Sapovirus (I,II,IV and V) 9 (4.0%), Rotavirus A 9(4.0%), *Giardia lamblia* in 7 (3.1%), Astrovirus 6 (2.7%), *E*. *Coli* O157 3 (1.4%), *Plesiomonas shigelloides* 3 (1.4%), Adenovirus F40/41 2(0.9%), *Vibrio* no cholerae 1 (0.5%) and *Yersinia enterocolitica* 1 (0.5%). The FilmArray did not identify any positive sample for *Entamoeba histolytica*, *Cyclospora cayetanensis* or *Vibrio cholerae* (Fig 1). When we analyzed the microorganisms found in co-infections, diarreicogenic *E*. *coli* were present in the context of a co-infection in more than 80.0% of the cases, whereas *Campylobacter* spp., *C*. *difficile* and *Cryptosporidium* were observed in less than 40.0% (Fig 1).

### Clinical-etiological characteristic and correlation

In total 74 patients had positive results only for bacteria (37.2%), 22 only for virus (11.0%) and 11 for parasites (5.5%). Patients with positive results were younger (40 vs 47 years, p = 0.024), sought medical attention earlier (4.9 vs 7.3 days, p = 0.0005), had more bowel movements per day (8.6 vs 6.7, p = 0.017), higher percentage of fever (44.3% vs 21.3%, p = 0.0017) and less comorbidities (69.6 vs 51.8%, p = 0.021) (Table 1). Polimicrobial infections were associated with a higher frequency of nausea (50.0% vs 32.0%, p = 0.046), abdominal pain (87.0% vs 44.0%, p<0.0001) and travel history (20.0% vs 5.0%, p = 0.0102) than those with only one pathogen detected (Table 1).

**Table 1:** Comparison of clinical characteristics between patients with positives vs negative results and mono vs polimicrobial infections.

Clinical characteristics	Positive	Negative	P value	Mono-microbial	Poli-microbial	P value
	N = 133	N = 66		N = 79	N = 54	
Feminine gender (%)	56	67	NS	58	52	NS
Median age (years)	40	47	0.024	42	39	NS
N° days of diarrhea at ER	4.9	7.3	0.0005	5.3	4.6	NS
Bowel movements per day	8.6	6.7	0.017	8.4	8.9	NS
Dysentery (%)	31.0	38.0	NS	18.0	30.0	NS
Fever (%)	44.0	21.0	0.0017	28.0	41.0	NS
Vomiting (%)	39.0	29.0	NS	26.0	35.0	NS
Nauseas (%)	56.0	52.0	NS	32.0	50.0	0.046
Abdominal pain (%)	88.0	82.0	NS	44.0	87.0	<0.0001
Dehydration (%)	64.0	64.0	NS	63.0	65.0	NS
Mild (%)	41.0	44.0	NS	44.0	37.0	NS
Moderate (%)	20.0	20.0	NS	16.0	24.0	NS
Severe (%)	3.0	0.0	NS	3.0	4.0	NS
Hospitalization (%)	44.0	51.0	NS	47.0	39.0	NS
Comorbidity (%)	52.0	70.0	0.021	59.0	40.0	0.036
Travel History (%)	11.0	7.0	NS	5.0	20.0	0.0102

As shown in Table 2, patients with bacterial infections had a higher frequency of dysentery (41.0% vs 18.0%, p<0.0001) and abdominal pain (95.0%, p = 0.027) compared to parasitic and viral infections respectively. Fever, hospitalization requirement and travel history were also more frequently associated with bacterial infection but it did not reach statistical significance. On the other hand, patients with viral infections presented a higher frequency of nausea (77.0%, p = 0.048) and vomiting (59.0%, p = 0.044). Higher number of bowel movements per day associated with more dehydration was also observed in viral infections but it did not reach statistical significance. No dysentery was observed in viral infections (Table 2).

**Table 2:** Clinical characteristics according to etiology.

Clinical characteristics	General	Bacteria	Virus	Parasites	*P value*
	N 199	N 74	N 22	N 11	
Feminine gender (%)	59	57	64	36	NS
Age mean (years)	43 (18–100)	42	42	35	NS
N° days with diarrhea	5.8 (SD 4.4)	5	4.7	6	NS
N° bowel movements per day	8.2 (SD 5.4)	7.7	9.4	6.5	NS
Dysentery (%)	33.1	**41.0**	**0.0**	18.0	<0.0001
Fever (%)	36.7	54.0	36.0	27.0	NS
Vomiting (%)	35.8	**32.0**	**59.0**	36.0	0.044
Nausea (%)	54.8	**51.0**	**77.0**	45.0	0.048
Abdominal pain (%)	86.0	**95.0**	**77.0**	91.0	0.027
Dehydration (%)	64.0	66.0	73.0	45.0	NS
Mild (%)	42.2	42.0	59.0	18.0	NS
Moderate (%)	19.6	20.0	14.0	27.0	NS
Severe (%)	2.0	4.0	0.0	0.0	NS
Hospitalization requirement (%)	46.2	51.0	32.0	36.0	NS
Comorbidity (%)	58.3	53.0	55.0	45.0	NS
Travel history (%)	10.0	15.0	9.0	0.0	NS

Finally, those patients with immunosuppression were found to have a higher frequency of positive results with parasites (23.0% vs 8.0%, p = 0.04) compared to immunocompetent patients, and showed a higher percentage of hospitalization (68.0% vs 44.0%, p = 0.04) (Table 3).

**Table 3:** Clinical characteristics according to immune status.

Clinical characteristics	Immunocompetent N, (%)	Immunosupressed N, (%)	*P value*
Total patients	177	22	
Positive results	116 (66.1%)	17 (77.2%)	NS
Bacteria	88 (49.7%)	11 (50.0%)	NS
Virus	37 (20.9%)	5(22.7%)	NS
** Parasites**	**14 (7.9%)**	**5(22.7%)**	**0,04**
1 microorganism	68 (38.0%)	11(50.0%)	NS
≥2 microorganisms	48 (27.0%)	6(27.2%)	NS
Median age (years)	43	45	NS
Comorbidity	93 (52.5%)	22(100%)	<0.0001
Dehydration	115(64.9%)	11(500%)	NS
Mild	77(43.5%)	6(27.2%)	NS
Moderate-severe	39 (22%)	5(22.7%)	NS
Dysentery	58 (32.7%)	7(31.8%)	NS
Fever	65 (36.7%)	8(36.3%)	NS
Hospitalization	77 (43.5%)	15(68.2%)	0,04
Travel History	17 (9.6%)	2(9.0%)	NS

Immunosuppressed: HIV (+), hematological malignancy, solid organ transplant, immunosuppressive therapy, chronic renal terminal disease

NS: Not significant.

We analyzed the clinical characteristics per microorganism considering only those patients with monomicrobial infections. Infections by *Shigella*/EIEC, *Salmonella* spp., and *Campylobacter* spp. were associated with a higher percentage of dysentery (67.0%, 50.0%, and 43.0% respectively), fever (56.0, 75.0 and 86.0%) and abdominal pain (100% in all cases) compared to the total. Patients with *C*. *difficile* infection were found to require more hospitalization (69.0%) and were more frequently females (85.0%). Patients with viral infections did not have dysentery and in the case of Norovirus and Astrovirus infections no patients required hospitalization. All patients with Sapovirus infection referred abdominal pain and Rotavirus was associated with a higher frequency of stool passages per day, dehydration and hospitalization requirements.

Bacterial infections occurred without a seasonal distribution with the exception of *Salmonella* spp. which showed a clear predominance during the summer months. In the case of viral infections, predominance was observed during the autumn–winter months compared to the spring- summer season (41.0% vs 21.0%). We did not observe differences in the case of parasites with the exception of *Giardia lamblia* that was detected more frequently during the spring-summer months. Statistical analysis was not performed for each pathogen due to the low number of cases.

## Discussion

To our knowledge this is the first study to characterize the etiological and clinical presentation of acute diarrhea in adults by a multiplex PCR in our country. The FilmArray is an FDA approved panel for the rapid detection of 22 pathogens in 1 hour. It is rapid and easy to manipulate eliminating the need of a specialized laboratory. Moreover, published data show excellent sensitivity and specificity in the detection of the pathogens included, increasing the number of positive results compared to the traditional laboratory methods (i.e. stool culture, microscopy, EIA) [19–20]. In our study, 66.8% of the samples were positive for at least one pathogen. This number is higher compared to what is expected from a standard stool culture [20, 21]. A study published by Stockmann et al. [22] compared the yield of the traditional microbiologic study compared to multiplex PCR observing 46.0% of positivity for the traditional methods compared to 65.0% with the multiplex PCR. This is explained by a better sensitivity of the PCR and to the extended number of pathogens included [23]. Many of these pathogens are either not included in the traditional study or not originally requested by the attending physician. The sensitivity of the culture of *Salmonella* spp., *Shigella* spp. and *Campylobacter* spp. when compared to PCR confirmed samples ranges from 66.6 to 76.9% [24]. The FilmArray panel has a comprehensive menu of pathogens that can be detected. In our institution, only *Salmonell*a spp. and *Shigella* sp. are routinely detected in the stool culture with the exception of *E*.*coli* O157 in patients younger than 10 years of age. Special cultures are available upon request for *Campylobacter* spp. and *Yersinia* sp. We observed positive results for 19 of the 22 target pathogens included, with the exception of *Entamoeba histolytica*, *Cyclospora cayetanesis* and *Vibrio cholerae*, microorganisms not endemic in our country. As such, a traditional stool study would have missed more than 75% of the microorganisms observed. Bacteria predominated over viruses and parasites. The most frequently detected microorganisms were diarrheagenic *E*. *coli* (especially enteropathogenic *E*. *coli*) followed by *Campylobacter* spp, *Salmonella* spp, *Clostridium difficile* and *Shigella/*EIEC. In the case of viruses, Norovirus was the most frequently observed microorganism, followed by Sapovirus whereas *Cryptosporidium* was the most frequently found parasite. This pathogen has been traditionally associated with infection in immunocompromised and pediatric patients and its prevalence varies according to the population studied. In our study, only 3 of the 11 patients with *Cryptosporidium* presented immunosuppression. Multicenter studies using multiplex PCR in low risk population showed frequencies from 2.0 to 3.0% [11, 25]. Thus, the importance of *Crysptosporidium* as a pathogen of acute diarrhea in immunocompetent adults could be underestimated due in part, to a lower sensitivity of the traditional methods [23–25].

Finally, 40.6% of the patient with positive result (27.1% of the total) showed more than one pathogen. The importance of polimicrobial infections has been reported previously. Published data estimates a frequency of 31.5% in the United States of America and 30.0% in Europe [11,25]. Recently published data showed 48.1% of co-infections in a private center in Chile, similar to our findings [26]. In most of the co-infections bacteria were involved, with only 2 patients with two viruses detected and no cases with more than one parasite. It is important to emphasize that the most frequently involved microorganisms in polimicrobial infections were diarreicogenic *E*. *coli*. Furthermore, 80.0% the *E*. *coli*´s were found in the context of a co-infection. In our study, patients with more than one pathogen did not differ in severity compared to those with monomicrobial infection with the exception of increased abdominal pain and nausea. Interestingly, the application of a multiplex PCR to cases and control subjects showed no statistical differences in the frequency EPEC and EHEC detection [20]. Thus *E*. *coli* pathogenic´s role could be debatable and more studies are needed. In the same way, the clinical relevance of polimicrobial infections requires further analysis.

The use of multiplex panels has given us invaluable information regarding the epidemiology of acute diarrhea in adult patients visiting an emergency room in a developing country. It has reinforced the importance of bacterial infections as well the role of parasites. As such, the strategies applied in our laboratory have been evaluated, considering for example, the search for *Campylobacter* in the routine stool culture. It is important to note that patients included in this study had moderate to severe diarrhea as such, this information can only be applied to this population and cannot be necessarily extrapolated to mild cases where a microbiology study is not routinely recommended. A rapid and sensitive result could allow the rapid instauration of an appropriate treatment. Nevertheless, its impact on clinical decision, cost-effectiveness especially in resource limited countries and the real pathogenic role of some the pathogens needs to be further investigated.
